# Built-up area and population density: Two Essential Societal Variables to address climate hazard impact

**DOI:** 10.1016/j.envsci.2018.10.001

**Published:** 2018-12

**Authors:** D. Ehrlich, T. Kemper, M. Pesaresi, C. Corbane

**Affiliations:** Joint Research Centre, European Commission, Via Enrico Fermi, 2749, I-21027 Ispra, Italy

**Keywords:** Human settlements, Built-up, Population density, Essential Societal Variables, Essential Climate Variables, Climate hazards

## Abstract

Scientists use Essential Climate Variables to understand and model the Earth’s climate. Complementary to the Climate Variables this paper introduces global built-up area and population density, referred to as Essential Societal Variables, that can be used to model human activities and the impact of climate induced hazards on society. Climate impact scenarios inform policy makers on current and future risk and on the cost for mitigation and adaptation measures. The global built-up area and global population densities are generated from Earth observation image archives and from national population census data in the framework of the Global Human Settlement Layer (GHSL) project. The layers are produced with fine granularity for four epochs: 1975, 1990, 2000 and 2015, and will be updated on a regular basis with open satellite imagery. The paper discusses the relevance of global built-up area and population density for a number of policy areas, in particular to understand regional and global urbanization processes and for use in operational crisis management and risk assessment. The paper also provides examples of global statistics on exposure to natural hazards based on the two ESVs and their use in policy making. Finally, the paper discusses the potential of using population and built-up area for developing indicators to monitor the progress in Agenda 2030 including the Sustainable Development Goals (SDGs).

## Highlights

• This paper introduces two essential societal variables (ESV) – global built-up area and global population density.
• The two ESVs are the building blocks for quantifying the human societal system as they measure the human presence on Earth.
• The two ESVs complement the essential climate variables in modelling climate impact on societies.
• The two ESVs are already used in disaster early warning systems, within disaster risk models, in system of indicators and in crisis management.
• The two ESVs are also tested for use in system of indicators used to measure progress towards the 2030 Development Agenda.

## 1 Introduction

Climate impact on human society can be modeled using a combination of Essential Climate Variables (ECV) (Bojinski et al., 2014) and Essential Societal Variables (ESV) that describe the human system. The ECV are the core set of variables used to characterize the Earth’s climate. ECVs are used to measure progress towards the objectives and the mandates of the United Nations Framework Convention on Climate Change (UNFCCC) and the Intergovernmental Panel on Climate Change (IPCC). The UNFCCC calls for an analysis of the impact of climate change on the human system (that includes settlements as well as the agricultural land, water resources and environmental assets that provide resources and deliver services) and of “mitigation and adaptation measures, to assess risks and to enable attribution of climatic events to underlying causes, and to underpin climate services” (Bojinski et al., 2014). The built-up area and the population density are the two ESV used to quantify what we are trying to protect from the impacts of natural hazards.

Societal variables are needed to analyze the human societal system. We introduce the term human societal system to indicate the integrated system that combines human activities and earth system processes (NASA Advisory Council, 1988; Steffen, 2005). The term includes what is referred to as human natural system integration (Liu et al., 2015, 2007; Noble and Huq, 2014), socio-ecological systems (Young et al., 2006) or coupled human environmental systems analysis (Liu et al., 2015). The term societal system aims to stress that human societies use materials and minerals, environmental assets and services, and are also producing artificial substances (such as nitrogen in the nitrogen fixation process) that affect the biogeochemical cycles, which ultimately modulates changes in climate processes. In fact, human societal activities are also categorized and referred to as socio-economic activities (Haberl et al., 2011; Steffen, 2005) or societal metabolism (Fischer-Kowalski, 1998; Haas and Andarge, 2017; Sorman and Giampietro, 2013) and are studied at local and national scale to assess sustainability of cities (Hoornweg, 2010; Johansson et al., 2012) or nations (Baccini and Brunner, 2012) and at global scale to assess planetary boundaries of earth system processes and global sustainability (Rockström et al., 2009).

We refer to the global built-up area and global population density as ESV for at least three reasons. First, the global population density layers can quantify globally the spatial extent of human presence on Earth, and are used to measure societal impact on climate models at local and global level. Second, the two variables are used to generate global exposure to hazards: essential information for use in crisis management to assess natural hazard risk globally (UNISDR, 2015a) also within the climate change community (Cardona et al., 2012). Urbanization as an accelerated physical phenomena of city and settlement expansion (Seto et al., 2014) is in fact a major risk factor. Third, the two variables can also be used as digital data infrastructure that uses the geographical location of the built-up area or of the population as spatial reference to add additional information. For example, when age structures, human development, poverty or income are combined with population density they can be used to characterize people’s vulnerability to a given hazard. Similarly, when the structural characteristics of the building stock are combined with location and size of built-up areas they can be used to define physical vulnerability to a given hazard. The built-up area and population with augmented information can thus be used to better model human societal activities that may include energy use and emissions. In this paper we refer to Essential Societal Variables to global built-up area and population density, unless otherwise specified.

This work focuses on ESV used as exposure to fast-onset climate related hazard – including cyclones, winds, floods, sea level surge. We address these selected hazards also because corresponding global hazard datasets were made available through the Global Assessment Report (UNISDR, 2015a) and could be used to quantify globally the exposure of built-up area and population. Climate hazard also includes heat waves, cold spells and droughts that may impact on people’s health or the food base that can be analyzed with datasets not available within this research and thus outside of the scope of this paper. The aim is not to be exhaustive in listing all climatological related hazards and their interaction as analyzed in (Revi et al., 2014) but rather to describe global built-up area and global population density that can be impacted by those hazards. Their vulnerability – the degree to which they will experience harm as a result of the impact of the hazards (Cardona et al., 2012; Turner et al., 2003), which is related to the characteristics of the built-up area or of population – is not addressed in this paper for the following reasons. Vulnerability of built-up area and population is always hazard specific (Schneiderbauer and Ehrlich, 2006) and location specific, and for which it is difficult to get data globally. It is generally assessed locally or at national level, and requires a range of input data that are outside of the scope of this paper. For example, physical vulnerability requires information on the structural characteristics of the building stock, which is derived from national databases (Dell’Acqua et al., 2013). Population characteristics that in large part define vulnerability are derived from national censuses that are not always available or from expensive field surveys covering only selected regions of the world (Tatem, 2015).

**Fig. 1:**
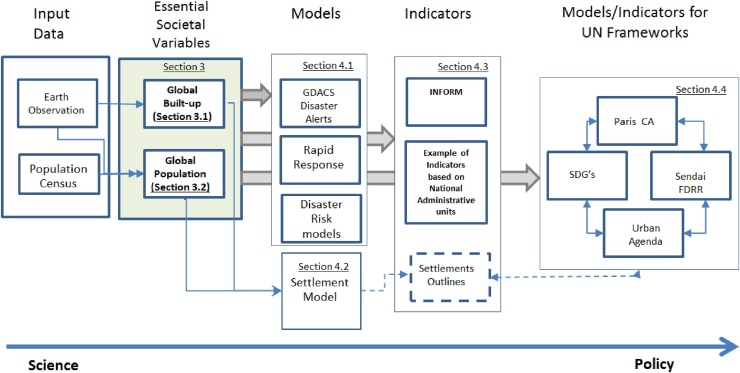
The Science-Policy continuum illustrates the production of built-up area and populating data and their use at different levels of aggregation in models, indicators and indicators systems used by policy makers. The shaded boxes describe the production of the variables at the core of this paper. The remaining boxes illustrate their use in disaster risk management services like the impact model Global Disaster Alert and Coordination System (GDACS), Copernicus Emergency Management Services (Copernicus-EMS), and the system of indicators including Index for Risk Management (INFORM), and United Nations Framework Indicator system.

This paper succinctly describes the production of the two global variables and their use. The paper first summarizes the production of the built-up area and population density and their characteristics. It then summarizes their use in modeling hazard impact and in systems of indicators for assessing humanitarian or disaster response needs. Fig. 1 illustrates the layout of the paper starting from the input data, the generation of built-up areas and population, their use in impact assessments and disaster risk models and finally their role in the production of indicators for monitoring the progress towards international frameworks such as the Paris Climate Agreements (United Nations Framework Convention on Climate Change, 2015), Sendai Framework for Disaster Risk Reduction (Sendai FDRR, UNISDR, 2015b); Sustainable Development Goals (UN General Assembly, 2015), and the New Urban Agenda (United Nations, 2016). This sequence highlights the generation of information products that can be directly used by scientists as well as policy makers at different stages; in models, indicators or system of indicators.

## 2 Essential variables and climate impact

**Fig. 2:**
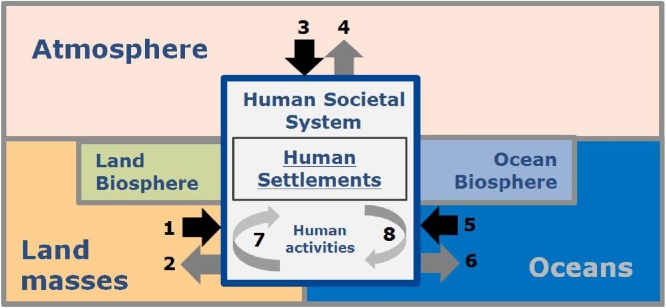
A simplified earth system model diagram showing climate sub-systems (based on GCOS 2016) with the human societal system and human settlement included. Grey arrows represent human activities that affect the climate subsystems. Black arrows refer to climate hazardous processes generated from climate subsystems (black) that impact society and settlements.

Essential Climate Variables have been extensively reviewed and described (WMO, 2016). Fig. 2 schematically describes the Atmospheric, Oceanographic and Terrestrial climate subsystems (GCOS, 2016) and the interaction with the human system through the societal-environment interaction processes. Human activity affects the biosphere of terrestrial landmasses and oceans from which it derives most of the energy, fibre, water and ecosystem services (Fig. 2, grey arrows). Societal demand for energy and materials is driven in large part by population expansion and the metabolism of society modulated by the economic system (Haberl et al., 2011). The demand for resources can be inferred in part by measuring the spatial extent of settlements, settlement metabolism as well as in land use changes (GCOS, 2015). Emissions, which are part of the undesired outcome of the use of resources, affect the climate variables (grey arrows 2, 4, 6) and in turn, the climate induced hazards.

The climate related fast onset hazards impact societies and their life supporting systems (Fig. 2, black arrows). The largest impact typically occurs within settlements as the destructive energy impacts the physical infrastructure and its resident population. Climate induced hazards include strong winds associated with tropical cyclones or tornados originated from atmospheric processes (arrows 1), storm surge from the oceans generating coastal floods that may be aggravated by climate induced sea level rise (arrow 3) and inundations and flash floods from terrestrial processes (arrow 5). Human activities can also generate man-made hazards that impact society (i.e. technological hazards; arrows 7 and 8) or amplify the outcome of disasters caused by natural technological hazards (Krausmann and Mushtaq, 2008). Slow onset hazards, including droughts, affect water supplies and other natural services, and impact population in the affected areas. This paper aims to quantify global built-up areas and population in human settlements that are exposed to fast-onset climate induced natural hazards.

The climate variables (describing climate processes) and societal variables (describing human activities) must be collected globally at fine geographical scale. In fact, “The climate system may be global in extent, but its manifestations – through atmospheric processes, ocean circulation, bioclimatic zones, daily weather, and longer-term climate trends – are regional or local in their occurrence, character and implications. Moreover the decisions that are or could be taken on the basis of climate change science play out at a range of scales, and the relevance and limitations of information on both biophysical impacts and social vulnerability differ strongly from global to local scale, and from one region to another. Explicit recognition of geographical diversity is therefore important for any scientific assessment of anthropogenic climate change” (Hewitson et al., 2014). That fine granularity is even more important in the measurement of the human dimension of climate change as settlements are covering only a small fraction of the surface of the Earth and hazard impact is always local.

Human settlements are the outcome and centre of activity of the human system, and host what is most valuable to societies (Fig. 2). Natural hazards impact is best understood by crossing hazard with settlement information (Pesaresi et al., 2017), for a number of reasons. First, the physical infrastructure of the settlement (the built-up area) and its population is what we are trying to protect. Second, both variables contribute to define exposure to hazards that in crisis management is used to quantify and model disaster risk and hazard impact. Third, the spatial detail of the built-up area “anchors human activity to the land” and provides the spatial reference that can be used to standardize in space and time other variables including carbon or energy emissions. Fourth, built-up area and population density can be used to partition the built-up space in settlement types such as towns, cities, megacities. The spatial extent of settlements can be used as geographical reporting units for generating settlement statistics as required in SDG reporting.

## 3 Human settlements and essential societal variables

The built-up area and population density layers are derived from two sources of input information, satellite imagery and population data available from censuses. The two ESV are described in subsections below.

Satellite imagery is the main information source used to extract the built-up areas; this was done as follows. The dominant physical settlement features that can be identified or inferred from imagery are constructed structures in the form of buildings or civil works (Pesaresi et al., 2013). Buildings are roofed and walled built structures (Merriam-Webster, 1989) used for shelter or for performing human activities (Pesaresi et al., 2013). This definition of building coincides with that of the INSPIRE directive (European Commission, 2007) with the exception that it also includes refugee camps, informal settlements, slums and other temporary settlements (INSPIRE, 2013). The location and the spatial size of buildings defines a planimetric surface area – referred to as building footprint area – that is the minimum structure or object to be measured. For this research project the information extracted from satellite imagery over the built environment –the physical space used for human habitation (Douglas, 1994) – is referred to as a built-up area when a pixel contains footprints of buildings or parts of buildings (Pesaresi et al., 2013).

### 3.1 Global built-up area layers from earth observation data

Global built-up layers have been produced largely using satellite Earth observation data. Built-up information is available also from aerial photography, cadastre databases and field surveys that are not addressed here as these data are not collected systematically across the entire globe and not with the same observation characteristics. Satellite earth observation is used for its synoptic view and regular repeated cycle of recording images that are comparable in space and time.

The built environment has been mapped from satellite data at different spatial resolutions (Gamba and Herold, 2009). Earlier global urban mapping studies used low to moderate resolution (300 m–1000 m spatial resolution) earth observation data (Potere and Schneider, 2007). The estimates of urban areas from these studies varied significantly (Schneider et al., 2010) in part due to the use of input satellite imagery at different spatial resolution. The changes in the extent of the global built environment have also been attempted using DMSP/OLS night time lights (Sutton et al., 2001). At regional level DMSP/OLS together with SPOT-VGT data were used to detect changes of settlement between 1998 and 2008 in India by (Srinivasan et al., 2013). MODIS 500 m resolution images were used to map urban areas in East Asia from 2000 to 2010 (Mertes et al., 2015). An extended review on land cover mapping is available from Ban et al. (2015).

The global built-up area layers used in this work are derived from processing the Landsat image archive (Pesaresi et al., 2016a), the most consistent satellite image dataset depicting the surface of the Earth. Landsat historical data are available as an open source data layer from United States Geological Survey (Markham and Helder, 2012). The data are available as geo-coded image products. The Landsat collection includes images from three Multi Spectral Scanners (MSS) with 75 m × 75 m, the Thematic Mapper 4–5 to 30 m × 30 m for the Enhanced Thematic Mapper (ETM) on Landsat 7, and to 15 m × 15 m for the Operational Land Imager (OLI) and Thermal Infrared Sensor (TIRS) on Landsat 8.

The Landsat bands maintain a consistent viewing geometry with stable Instantaneous Field Of View (IFOV), referred to herein as spatial resolution of imagery (Woodcock and Strahler, 1987). That stable viewing geometry and viewing precision is a fundamental image parameter for detecting and mapping small features such as building structures that are found in human settlements.

The classification approach adopted is based on Symbolic Machine Learning (SML) methodology (Pesaresi et al., 2016b). SML is based on image data quantization sequencing and association analysis. The image data sequencing aims to reduce the number of features to process. The association analysis links the set of reference data that acts as training data with the feature space used in the sequencing. The association is measured as a confidence index that expresses the association between the image data layers and the reference data. Values close to 1 indicate that the feature sequence is strongly associated with the image class of interest – the built-up in our case – while values close to −1 indicate that the feature is strongly associated with the classes other than built-up.

This classification generates four built-up area layers corresponding to the epoch 1975, 1990, 2000 and 2015. The layers are available at a minimum mapping unit corresponding to the spatial resolution of Landsat, 38 m × 38 m. For modelling purposes the built-up area layers are also aggregated at 1 × 1 km grid cell size and made available and referred to as built-up spatial grids. Each 1 km × 1 km cell estimates the density of built-up within the cell. The built-up spatial grids are also available for the epoch 1975, 1990, 2000 and 2015.

**Fig. 3:**
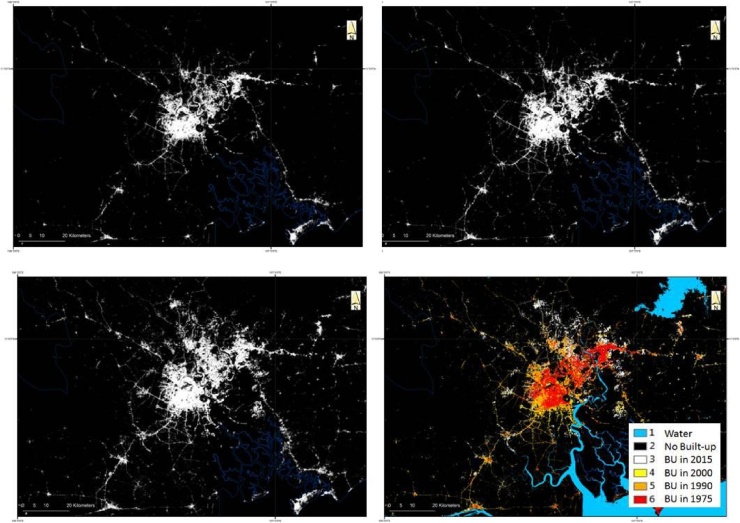
Ho Chi Min City in 1990 (a), 2000 (b), 2015 (c) and the combination of the above epochs in colour coded as mapped from Landsat imagery. The temporal datasets is particularly well suited to the analysis of exposure to natural hazards such as floods.

**Fig. 4:**
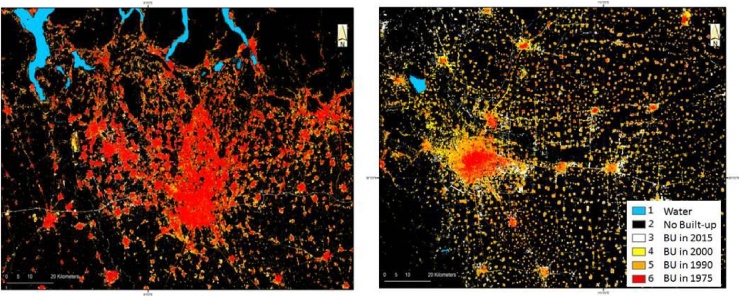
Northern Italy centred on the City of Milan and Eastern China centred on the city of Shi Jia Zhuang (China) over an area of 150 × 120 km showing two different urbanization and urban growth patterns.

The global built-up area layers contain information that is standardized in time and in space. It it enables comparison of spatial growth and changes in built-up area both in time and space at different scales (Pesaresi et al., 2016c). For example, Fig. 3 provides an example of built-up area maps for the city of Ho Chi Min city for year 1990, 2000 and 2015, and a multi-temporal map that includes also information on the built-up areas in 1975. Similar multi-temporal maps can be replicated for any city or settlement in the world (Fig. 4).

Information on the built-up area helps us to form a better understanding of patterns of urbanizations across the globe and the percentage of built-environment over other land uses and to derive indicators and statistics. Global urbanization patterns as well as indicators including the built-up land per person that can be computed globally from the built-up area and population density layers and addressed in Melchiorri et al. (2018). The Atlas of Human Planet 2017 (Pesaresi et al., 2017) illustrates the extent of built- up in hazard-prone areas. This is further discussed in Section 4.3.

### 3.2 Global population density layers

As census information is typically released with spatial units corresponding to national or sub-national administrative units, it is too coarse to be used for analyzing human activities or hazard impact including disaster risk assessments. For that reason, global population densities are produced using population census data, models and variables that allow for the spatial disaggregation of the population figures at fine scale spatial units typically based on spatial grids. Earlier work spatially disaggregated population data available from censuses using geo-spatial models (Tobler et al., 1995). Later, the variables for the disaggregation included land cover information and elevation (Dobson et al., 2000) and field data collected from household surveys (Stevens et al., 2015).

The global population density described in this work is produced from a combination of built-up area and population data available at administrative boundaries from the Gridded Population of the World (GPW) project (Center For International Earth Science Information Network-CIESIN-Columbia University, 2017). The GPW project assembles, from a number of data providers, a global population database that combines spatially explicit administrative boundary and corresponding population estimates for use in population disaggregation (Balk et al., 2006). The GPW global population database available as administrative boundaries is disaggregated spatially in this research based on the 1 km × 1 km built-up spatial grid. The built-up spatial gird is used to “anchor people to the ground”. This is based on a probability that is modified by combining the built-up information with population suitability (Freire et al., 2015). The population density layer is made available as a global grid of 1 km × 1 km spatial grids and available for the four epochs 1975, 1990, 2000, and 2015.

## 4 Built-up area and population density in models and indicators

Variables used to model human activities are typically collected at fine spatial scale in order to capture the high diversity of land use. For example both impact and disaster risk models use high spatial detail human settlements data to match the spatial detail of hazard information layers. These measures of exposure to hazards are then used for developing, formalizing and calibrating the models that aim to describe disaster outcomes and to understand trajectories of land degradation or risk accumulation as used by decision makers (Di Baldassarre et al., 2017). Examples of crisis management services that use population and built-up in modelling disaster processes are briefly described in Section 4.1.

Built-up area and population density can also be used to build indicators, an aggregated measure of one or more variables in support of policy making. Indicators are typically computed based on geographical reporting units coinciding with administrative boundaries at national or sub-national level. The indicators provide aggregated information of the state of the human activities including urban spatial growth and their historical trends. Indicators are used by policy makers to allocate resources and to understand progress in the implementation of policies and their impacts. Indicators are also used to document potential future impact of natural hazards as briefly addressed in Section 4.3.

Finally, the use of population density and built-up area density for monitoring targets set by international framework agreements is briefly summarized in Section 4.4. Some SDG indicators also require the definition of urban areas. For that purpose the human settlements model (Dijkstra and Poleman, 2014; Peasresi et al., 2016) can generate settlement outlines that complements those produced from administrative boundaries as addressed in Section 4.2.

### 4.1 Built-up area and population density in modeling hazard impact

Built-up area and population density are used in impact and risk assessment models and within the indicators system of disaster risk reduction frameworks (i.e. the Sendai Framework for DRR). In fact, population location is the primary exposure variable in any disaster risk analysis, alert or impact model. Casualties are the main indicator of the severity of impact and used as a target indicator in the Sendai Framework for Disaster Risk Reduction. The built-up area is a key exposure variable for measuring the impact exerted by all fast-onset hazards on the built environment.

The Global Disaster Alert and Coordination System (GDACS) [http://www.gdacs.org/] is an automated system that provides early warning and a preliminary impact assessment on natural hazards around the world. It is a free system that requires only registering to receive the alerts, and is widely used within the humanitarian aid community. The alerts are issued for earthquakes and possible subsequent tsunamis, tropical cyclone, floods and volcanic eruptions. The impact assessments are generated based on the severity of the hazard and the potentially affected population. The finer and the more precise the population information the better the impact estimates.

The Copernicus Emergency Management Service (Copernicus EMS) [http://emergency.copernicus.eu/] provides spatial information on built-up and population presence for emergency response for different unfolding disasters due to meteorological hazards, geophysical hazards, deliberate and accidental man-made hazardous events and other humanitarian emergencies. Copernicus EMS delivers fine scale built-up maps with hazard extents and an assessment of the damage severity. The damage assessment maps may also provide relevant and up-to-date information that is specific to affected population and assets, e.g. settlements, transport networks, industry and utilities. The techniques of built-up information extraction used may in the future also be able to provide local built-up maps based on the finest scale open source satellite imagery. The built-up area and population density data are essential societal variables in probabilistic risk models. Probabilistic risk models are the most important tools at the service of national authorities to estimate future losses due to natural hazards. In fact, for fast onset hazard, the built-up complemented with its vulnerability and the value of the assets is used to estimate the probable total losses for a time span in the future. Population data are needed for estimating the potentially affected people while population and empirical vulnerability functions are used to calculate average annual human losses (Silva et al., 2014; UNISDR, 2015a). The Global Assessment Report for example has estimated future losses of earthquakes, floods, cyclones, sea level surge and tsunami based on a combination of global hazard information layers available for a given return period and global population density data used as exposure (UNISDR, 2015a).

### 4.2 Built-up area and population density for classifying human settlements

Built-up area and population density are used to classify human settlements based on their size using a Human Settlement Model. The Human Settlement Model consists of a set of geo-spatial rules used to partition the global built environment into three settlement types: urban centers, urban clusters and rural areas. The model builds on the “harmonized definition of cities and rural areas” (Dijkstra and Poleman, 2014) devised for a database of population densities using the administrative unit as spatial reference. In this work the same geo-spatial rules are applied to the built up area and population density datasets with spatial units of 1 km^2^. Each cell is classified as urban centers, urban clusters and rural areas. Urban centers include grid cells with more than 1500 people and contiguous cells that make up more than 50,000 people. Urban clusters include grid cells with less than 1500 people but no lower than 300 people and contiguous urban cells that account for 5000 people. Rural areas include grid cells with less than 300 people (Peasresi et al., 2016).

The Human Settlement Model was developed to address settlement boundaries as “Any empirical analysis of urban and rural areas, as well as human settlements, requires clear delineation of physical boundaries” (Seto et al., 2014). The settlement classification aims to improve our understandings of human settlements globally in two ways. First, it uses a standardized criterion to define settlements across the globe, and it uses the same spatial unit, the 1 × 1 km grid cell, that makes the settlement model spatially and temporally consistent. In fact, the lack of consistency in data was one of the drawbacks in global settlement analysis. Second, it proposes an alternative to the urban and rural classification used by censuses worldwide that is now considered inadequate to describe current urbanization and spatial growth processes (Morrill, 2004). In fact, urbanization for most cities now extends beyond the administrative municipal boundaries or city proper and the new built-up agglomeration transcends the jurisdictional scales (Cash et al., 2003) at which urbanization is typically reported (Montgomery, 2008). The urban centers computed provide a new insight on the number and geographical distributions of cities worldwide. The spatial geographical unit can be used for measuring SDG indicators and for addressing the risks of climate induced hazards in urban areas.

### 4.3 Built-up area and population density for indicators

The impact of natural hazards on society can also be assessed in the form of indicators. For example, INFORM (De Groeve et al., 2014) is a global, open-source risk assessment index for humanitarian crises and disasters. It is based on a set of indicators measured at the national and subnational level. It uses a wide variety of indicators including those related to natural hazards and disasters. The indicators are based on the intersection of hazard maps and population densities. Some of the hazard indicators used in INFORM include changes in exposure as described below.

**Fig. 5:**
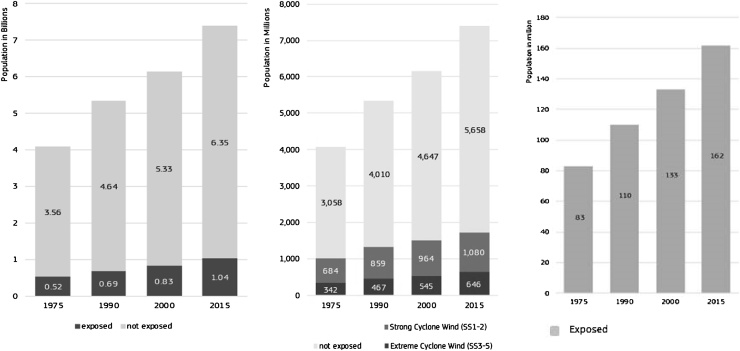
Share of global population exposed to floods (left) with 100 years return period; to cyclone winds (centre) and total population exposed to sea level surge (right) with 250 years return period.

Exposure and the increase in exposure to natural hazards are major risk drivers. Fig. 5 shows changes in built-up area and population from 1975, 1990, 2000, and 2015 for flooding, cyclone winds and sea level surge. All three hazards may increase their frequency and intensity due to a changing climate. The figures are produced by intersecting hazard layers made available from the Global Assessment Report (GAR, UNISDR, 2015a) and of the Global Ensemble Streamflow Forecasting and Flood Early Warning (GLOFAS, Alfieri et al., 2013). The statistics produced at country and continental scale are summarized in (Pesaresi et al., 2017). The data also allow for local analysis. All hazards are different as their impacts are generated from different physical processes. Flooding affects more people than any other hydro-meteorological hazard including that of cyclone winds (Fig. 5). Changes of exposure over time, when measured globally follow the trend of global population growth, and future work will address the local analysis of exposure in hazard hotspot areas.

Re-drawn from (Pesaresi et al., 2017).

**Fig. 6:**
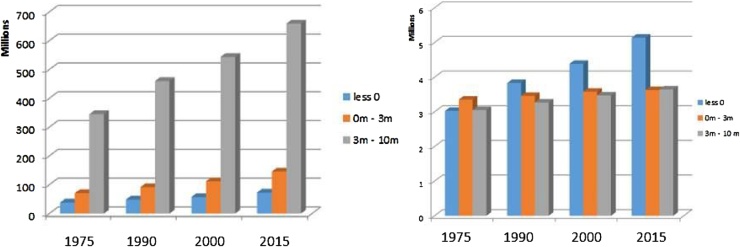
Population of the world living in 3 elevation classes: areas below sea level, between sea level and 3 m, and between 3 and 10 m (left). Population of The Netherlands living in the same three elevation classes (right).

The threat of sea level rise due to climate change is also a concern for policy makers. Figure 6shows changes in exposure over time in low elevated coastal areas that are at risk to sea level rise. In fact, the areas below sea level are already protected with infrastructure including dams and gates that will need to be reinforced and expanded in a sea level rise scenario (Hallegatte et al., 2013). Other unwanted impacts of sea level rise include salinization of water table and increased erosions (Wolters and Kuenzer, 2015) that are more difficult to mitigate.

The threat of sea level rise was assessed by analysing low elevation coastal areas calculated from the Digital Surface Model (DSM) derived from Shuttle Radar Topography Mapping mission (Farr et al., 2007). The DSM data were subdivided in four classes; elevation 1: the areas below sea level; elevation 2: that between 0 m and 3 m; elevation 3: that between 3 m and 10 m and elevation 4, elevation above 10 m. The first 3 classes were then crossed with the GHSL population data to show the changes in exposure in time. Examples of global statistics of low elevated coastal zones and that for one country are shown in Fig. 6 and a more complete discussion is available in (Ehrlich et al., 2017).

The work provides a more precise insight over population in low coastal areas. Less than 1% of the world population lives below sea level. That percentage has only slightly increased over time. The total population below sea level accounts in absolute terms to 74 million (2015) and has increased from 38 million in 1975. The total population in areas below 10 m accounts in absolute terms to 455 million in 1975, 879 million in 2015 (approximately 11% of total population). The Netherlands among all the countries of the world contains the largest share of built-up and population below sea level. For example, 74% of the built-up is in areas below 10 m elevation. The share of population living below sea level has increased from approximately 3 million to just over 5 million between 1975 and 2015. In addition, approximately 4.5 million live in areas located between 0 m and 3 m elevation.

### 4.4 Built-up area and population density for monitoring international frameworks

International framework agreements are governance processes aiming to steer society into sustainable and resilient trajectories, and typically rely on targets and indicators to measure progress. The Paris Climate agreement is directly linked to the Sustainable Development Goals (SDGs), New Urban Agenda and the Sendai FDRR. Table 1 summarises the possible use of global built-up and population densities in the four framework agreements as well as the overlap and links between the four frameworks.

**Table 1:** Use built-up area and population density in measuring SDG targets with a link to other framework agreements.

SDG Goals	SDG Target/Overall Aim	Link with other international frameworks	SDG indicators
Sustainable cities (11)	*Urban (settlement)* *resilience*	Sendai FDRR: Understand disaster risk (Priority 1) Invest in risk reduction for resilience (Priority 3)	1. Both Built-up area and Population density are “exposure “in the disaster risk equation. The settlement model can be used to define the spatial extent of urban areas as required in the development of the i*n*dicators that are required to report at city level
Sustainable cities (11) and Climate action (13)	*Urban (settlement)* *resilience* Increase number of cities addressing adaptation to climate change (11.b)	Urban agenda: strengthen the resilience of cities in line with Sendai FDRR (77)	2. The settlement model can be used to define the spatial extent of urban areas as required in indicator
		Paris Agreements: biannual indicative quantitative and qualitative information … on financial resources for mitigation and adaptations (Article 9: (5)	3. Mitigation and adaptation relies on financial estimates based on risk of future losses based also on changes in exposure
	Reduce number of deaths and economic losses (11.5)	Paris Agreements: Averting, minimizing and addressing loss and damage associated with the adverse effects of climate change (Art. 8)	4. In risk modelling future losses and damages are estimated with risk models that include exposure and change in exposure
		Sendai FDRR: Reduce global disaster mortality (target a) Reduce the number of affected people (target b) Reduce the cost of economic loss (target c) Reduce damage to critical infrastructure (target d)	5. All measures of losses would ideally use exposure to normalize loss trends related to the four targets (a-d). For example 100 fatalities (target a) weigh differently if they occur within an exposed population of 1000 or 1 Million.
	Enhance inclusive and sustainable urbanization (11.3)	Paris agreements: Strengthening the global response to the threat of climate change (Art 2)	6. In a changing climate, climate hazards need to be re-calculated and updated constantly and measures of exposure to the hazards need also to be re-calculated accordingly.
		Sendai FDRR: Build back better (Priority 4) Urban agenda: Long term urban territorial planning and spatial development (72) “build back better” in the post-recovery process (78)	7. Baseline information proposed by GHSL can be used to identify the highest built-up risk areas that need to be retrofitted or developed with appropriate risk adverse measures
		Urban agenda: Urbanization and land consumption is key to urban sustainability and energy efficiency	8. Indicator 11.3.1 Ratio of land consumption rate to population growth rate as an indicator to measure human impact on the Planet

Built-up area and population density can be used in different combinations with other data when building indicators. Aggregation of built-up and population density can be used to generate statistics on urbanization, land consumption in different countries, and percentage of urban dwellers over total population. Built-up area and population density may include attributes available from other sources, and address poverty or health as covered by SDG goals 1–5. For example, population changes over time can be used to model migration push factors in rural areas by modelling scarcity of land resources and societal demands. The precise spatial information provided by built-up area and population density is also important to address the sustainability of cities by modelling the access to resources at the local level and the ability of the environment to absorb its metabolic wastes in urban settings.

All SDG indicators are people and society centred, and most rely on measurements that either use population or built-up figures. All indicators will be calculated by authorities in each country for their territory with data they collect or provide. However, international institutions may provide global datasets that can be used to measure indicators independently and thus assess progress towards goals, and/or to supply countries with data for their territory that they are not able to produce. The discussion herein only addresses SDG 11 and SDG 13 and the explicit or implicit need for spatial measures on settlements with reference to reporting of the Paris Climate Agreements, Sendai FDRR, and Urban Agenda. Table 1 provides 8 examples where the two variables can be used. Examples 1 and 2 require the spatial extent of urban areas; in examples 3–7 the variables are used as exposure to hazards; and in example 8 the two variables are used directly in the development of the indicator on ratio of land consumption rate that can be computed at the local level.

Population and built-up area are essential baseline datasets for the Paris Climate Agreement that calls for an understanding and quantification of emissions, a reduction of non-renewable emissions and a response to the impacts the climate will have on societies. This is expressed as “Strengthen the global response to the threat of climate change, in the context of sustainable development and efforts to eradicate poverty (Article 2)” and “averting, minimizing and addressing loss and damage associated with the adverse effects of climate change (Article 8)”. In addition, the Paris agreements calls to “… biannually communicate indicative quantitative and qualitative information … on financial resources for mitigation and adaptation” (Article 9: Section (5)). In a changing climate, hazards change their geographical impact, and exposure, which is also changing, needs to be re-calculated accordingly. In fact, a rapidly changing built-up and population exposure in large parts of the world is the biggest driver to risk (UNISDR, 2015a).

The ESVs include the assets that are exposed to the impact of the hazard and linked directly to the priority and target of the Sendai FDRR. The main goal of the Sendai FDRR is to improve knowledge on disaster risk and to reduce risk. Gridded built-up and gridded population density defines the exposed assets that are one of the components of the risk equation and thus directly address Sendai Framework Priority 1 related to Understanding Risk. The GHSL multi-date built-up area is particularly suited to address also the changing nature of risk. GHSL built-up area also addresses Priority 3, build back better by providing the baseline information on which to construct an improved built infrastructure. In addition there is secondary use of the gridded built-up and population in measuring the Sendai FDRR targets related to mortality (a), affected people (b), and physical damage and losses including that of critical infrastructure (c, d). These targets need to be normalized with the exposure data collected over time to understand progress in disaster risk reduction.

The built -up area and population information are used in mitigation, adaptation and sustainable development strategies. In fact, SDG is about improving wellbeing of people and communities and society at large. That development will be measured in part from societal variables and in part from variables of the natural environment. Poverty, zero hunger and wellbeing can also be measured using population density that can be used to generate the demand of environmental services. These societal environmental interactions differ based on the type of economies. Subsistence economies rely more on the environmental services in the proximity of settlement, while more affluent economies outsource the production of food and energy to regions often very far apart. That relation is continuously modified by progress and technological innovation that facilitates the production and transport of food and commodities. The Different SDGs aim to measure processes occurring at the global scale, or at the regional or local scale. Goals 13, 14, 15 are addressing climate, land, and marine resources at the global scale. However, the demand for those resources is generated locally but its cumulative effect is then modelled globally.

The New Urban Agenda contributes to SDG 11 addressing sustainable cities and infrastructure that calls for long term urban spatial planning and territorial development useful especially to accommodate potential sea level rise. Planning and managing spatial development is one of the key areas of action that requires up-to-date information on built-up, population density as well as land use and cover and vulnerable areas that should be incorporated in the planning process, especially at local level. In fact, open and easily accessible geospatial data can support monitoring in many aspects of development, from health care to natural resource management. The New Urban agenda also calls for building resilience of urban communities and build back better, goals that in turns link with that of the Sendai FDRR.

## 5 Conclusions

Mitigation and adaptation to climate change require both essential climate variables coined by the climate change community as well as societal variables to quantify the human societal system as addressed herein. The human societal system operates based on societal activities and some are tightly coupled with the natural systems. In fact, the great challenge of addressing environmental and global sustainability is to be able to measure and model the earth system and its subsystems and to identify the processes and their development trajectories and the climate impact on societies.

Datasets to describe the societal systems and especially those that unfold at the global level are still in short supply. Human activities are mostly modelled at local scale even if many processes have an impact at global scale. These include urbanization (Revi et al., 2014), the global displacement of land use (Weinzettel et al., 2013), the looming land scarcity in an era of economic globalization (Lambin and Meyfroidt, 2011) and the dynamic of the food system (Ericksen, 2008), or even the water cycle (Trenberth and Asrar, 2014) that are all related to climate impact, mostly as potentially underlying risk factors (Jha et al., 2013). Societal variables are essential in the quantification of these processes.

This paper describes the global built-up area and global population density typically used to describe and characterize human settlements. The two variables have a global scope and yet the measurement is available at fine scale and suited to address global processes as well as local processes, including the impact of climate induced natural hazards. The information can be aggregated to the spatial unit of interest. The population density can be used in the future also to address slow onset climate hazards such as droughts that affect the nutritional and health status of people. In fact, the slow onset climate hazards need to be addressed within the wider analysis of land degradation and water scarcity as advocated in the food water energy nexus (Biggs et al., 2015).

Despite common belief, human activities are not well understood at global level, among other reasons for a lack of good assessment. Urbanization for example has not been quantified using rigorous measuring techniques. Patterns of urbanization and the way they influence disasters or the sustainability of resources are not very well captured by fragmented datasets. The global built-up areas and population density data are used to make up for the missing information in order to understand disaster impacts and to model mitigation and adaptation strategies for a potential different climate. As countries move forward in addressing the issue of climate change, with much of the focus on emission reduction targets and their challenges, the relationship of climate change and its hazards and the implications for human settlements around the world must be kept at the forefront of thinking and action (McBean and Ajibade, 2009).
